# Mechanical and Microstructural Characterization of Rammed Earth Stabilized with Five Biopolymers

**DOI:** 10.3390/ma15093136

**Published:** 2022-04-26

**Authors:** Alessia Emanuela Losini, Anne-Cecile Grillet, Monika Woloszyn, Liudmila Lavrik, Chiara Moletti, Giovanni Dotelli, Marco Caruso

**Affiliations:** 1 LOCIE, CNRS, Universite Savoie Mont Blanc, 73000 Chambery, France; anne-cecile.grillet@univ-smb.fr (A.-C.G.); monika.woloszyn@univ-smb.fr (M.W.); 2 Department of Chemistry, Materials and Chemical Engineering “Giulio Natta”, Politecnico di Milano, 20133 Milano, Italy; liudmila.lavrik@mail.polimi.it (L.L.); chiara.moletti@polimi.it (C.M.); giovanni.dotelli@polimi.it (G.D.); 3 Material Testing Laboratory, Politecnico di Milano, 20133 Milano, Italy; marco.caruso@polimi.it

**Keywords:** rammed earth (RE), bio-stabilizers, biopolymers, waste materials, unconfined compressive strength, microstructural characterization

## Abstract

This study aims to check the compatibility of a selection of waste and recycled biopolymers for rammed earth applications in order to replace the more common cement-based stabilization. Five formulations of stabilized rammed earth were prepared with different biopolymers: lignin sulfonate, tannin, sheep wool fibers, citrus pomace and grape-seed flour. The microstructure of the different formulations was characterized by investigating the interactions between earth and stabilizers through mercury intrusion porosimetry (MIP), nitrogen soprtion isotherm, powder X-ray diffraction (XRD) and scanning electron microscopy (SEM). The unconfined compressive strength (UCS) was also evaluated for all stabilized specimens. Three out of five biopolymers were considered suitable as rammed earth stabilizers. The use of wool increased the UCS by 6%, probably thanks to the combined effect of the length of the fibers and the roughness of their surfaces, which gives a contribution in binding clay particles higher than citrus and grape-seed flour. Lignin sulfonate and tannin increased the UCS by 38% and 13%, respectively, suggesting the additives’ ability to fill pores, coat soil grains and form aggregates; this capability is confirmed by the reduction in the specific surface area and the pore volume in the nano- and micropore zones.

## 1. Introduction

The building sector is responsible for a large contribution of greenhouse gas emissions, due to the extraction, processing and transportation of raw materials, as well as the energy consumed during the construction phase and the building lifetime. The direct impact of cement production alone was estimated at around 8% of the global CO_2_ emissions in 2014, due to the carbon oxidation and fuel consumption during the production phase [1]. Moreover, the additional emissions of the construction process should be taken into account, as well as the waste generation due to demolition at the end of life, which accounts in Europe for about 35% of the total waste generation [2]. In order to find eco-friendly alternatives for the building industry, traditional techniques of construction that exploit natural and locally available materials (clay, wood and straw) are nowadays attracting the interest of architects and engineers.

Clayey soil is widely present in all continents and regions, and where available it is obtained directly from the excavation of foundations, avoiding transportation costs and emissions due to the production of the binder. Due to the high variability of this material, the suitability of the earth should be verified before starting construction [3]. Moreover, raw earth is recyclable and reusable after the demolition, thanks to the absence of the firing process. The practice of reusing the material of the ancient wall is widely carried out and practiced, due to the possibility of disaggregating and moistening the earth to restore a building or raise a different edifice [4]. This process is possible for the unstabilized raw earthen buildings, where clay plays the role of binder between the bigger grains of silt, sand and gravels [5].

Among the numerous raw earth processing techniques, the present study focuses on rammed earth (RE) constructions. This technique is based on the compression of earth layer by layer into vertical formworks to create a wall. This material owes its strength to the compaction effort that enhances the mechanical strength of the material. The RE technique is widely spread in different parts of Europe and used in the Southern France, where it is possible to find entire villages built with this technique, locally called ‘pisé’. Common values of unstabilized RE density are between 1700 and 2200 kg/m^3^ with a range of mechanical strength of 0.4–3 MPa [6]. A compaction effort around 5–10 MPa is normally applied during the construction process, excluding some experimental methods of hyper-compaction which arrive at 100 MPa [7,8]. According to different guidelines, not all the soil typologies are suitable for RE techniques [3,9]. Therefore, the soil can require grain size distribution modification, to provide well-graded distribution and a minimum content of clay, usually indicated at around 5% for all the techniques [3]. A well-graded distribution is suitable to minimize the void ratio and to increase the grain cohesion, thus improving the mechanical strength and durability [10]. A roof with large overhangs, combined with a good foundation to isolate the wall from capillarity increases are necessary to assure durability and protection against weathering, preventing damage to the façade. Additionally, stabilization with lime or cement is the most common procedure to enhance both the mechanical and weather resistances, with weight percentages varying between 5 and 15% [11,12]. On the other hand, the stabilization with cement increases the environmental impact, compromises the recyclability of the earth and may reduce the hygroscopic properties of the material, and thus the hygrothermal performance of the building during its lifecycle [13,14,15]. Indeed, the hygroscopic properties of earthen materials allow a natural control of the indoor humidity, contributing to maintain the moisture in the suitable range for human health [16]. This phenomenon is caused by the absorption into the materials of molecules of water that can change phase and store or release heat, generating good thermal inertia and moisture buffer value.

With the aim of avoiding the use of cement while increasing the mechanical properties of the material, the present work investigates the application of different promising biopolymers for rammed earth stabilization, including their influence on the mechanical properties of the material. Indeed, fibers and biopolymers can offer an alternative that fits the circular economy requirement: maintaining recyclability and the use of industrial and agricultural by-products as natural stabilizers. In a recent review paper, the authors showed a selection of natural additives and biopolymers for raw earth stabilization, evidencing the large availability of waste and recycled materials suitable for this aim [17].

## 2. Materials and Methods

### 2.1. Rammed Earth

RE is the fundamental component of our study. In order to obtain an appropriate rammed earth, the soil named ABS (provided by the company Minerali Industriali S.r.l. by an Italian cave near Lozzolo VC, Piemonte, Italy) was mixed with washed sand from Piave river, supplied by company Dal Zotto S.r.l. (TV, Veneto, Italy). The sand was added in a proportion of 3:1 to the soil ABS, respecting the recommendation of the guidelines on the suitable clay content for RE construction (clay fraction between 5 and 16 wt%) [10]. To simplify the discussion, “MIX” will be used as an abbreviation to refer to the 1:3 soil-to-sand ratio preparation. The grain size distribution of the MIX has been chosen to have a maximum diameter of 2 mm in order to reduce the time required to prepare and condition the samples with smaller dimensions to execute the UCS test [18,19]. It has been reasonably assumed that this choice does not affect the possibility to investigate the effects of biopolymers on soil stabilization.

**Figure 1:**
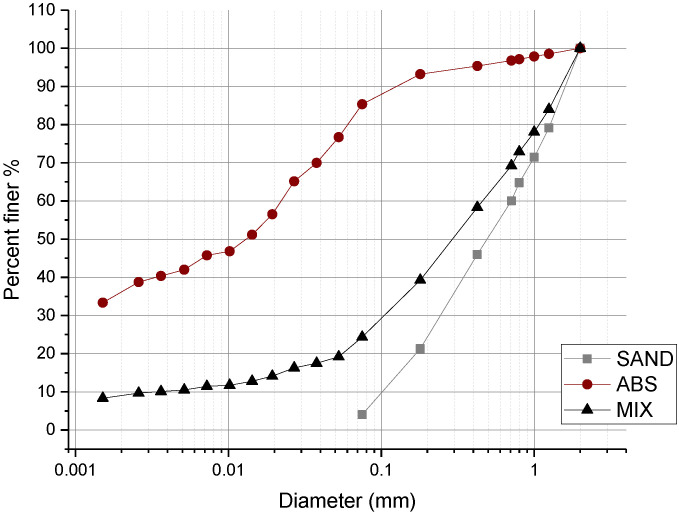
Grain size distribution: soil (ABS), SAND, and MIX (1:3 soil-to-sand ratio) [20]. Copyright 2021 Scientific.Net.

The grain size distribution obtained by the characterization tests on the ABS, the sand and the MIX are reported in Table 1 and Figure 1 [20]. The granulometric size distribution was determined by a hydrometer and by sieving, and the grain density was measured using the pycnometer method. The Atterberg limits were determined using the cone penetrometer method and the use of XRD analysis completed the characterization of the soil composition [21].

ABS soil is classified as silt (44%) with a small percentage of clay (36%) and sand (20%) [22]. XRD (X-ray diffraction) analysis confirmed that ABS soil contains an abundant presence of quartz albite (sodium feldspar), microcline (potassium feldspar) and has a mixed clay composition, with the presence of illite, kaolin, and a minor fraction of smectite. More details are reported in Section 3.6.

### 2.2. Additives

A large selection of natural additives and biopolymers tested for raw earth stabilization was presented in a recent review by the authors, including 50 independent studies [17]. This rich literature investigation enabled the identification of suitable biopolymers for the present research. In addition, the choice was determined by different criteria of environmental sustainability, recyclability and local availability of materials. Wool sheep fibers, lignin sulfonate and tannins were selected from previous literature research [17]. Citrus pomace was previously tested in a master project developed at Politecnico di Milano in 2019 [23]. This additive for earthen plaster preparation improved the adhesion strength and durability of the mixture [23]. Five biopolymers were selected to stabilize the MIX: citrus pomace, tannin, sheep wool fibers, lignin sulfonate and grape-seed flour. Lignin sulfonate and tannin are powders, while citrus pomace and grape-seed flour are residues of fruits and vegetables which contain a part of fibers. For this reason, they will be considered as fibers and compared to sheep wool fibers in the discussion of results.

The wide availability of grape-seed flour material and its low commercial value (0.065 EUR/kg) were considered interesting factors to investigate its suitability as a natural additive, but no literature provides evidence on the use of grape-seed flour as a stabilizer.

Citrus pomace is a common by-product of juice production, composed of peel and residues of citrus, which includes oranges, lemons, limes and mandarins. 30 million tons of citrus fruits annually produced are processed industrially, discharging almost 50% of wet fruit mass with low market value, that can be used for animal feeding or biofuel production with low commercial value (0.01 EUR/kg) [24]. The fresh material is highly biodegradable, and it is often dried to reduce the cost of transportation and storage. The material used for the test was kindly provided by the company Silvateam S.p.a (Bergamo, Italy), dried for 24 h at 50 °C and meshed to 1 mm to facilitate the dispersion when mixed with soil.

Sheep need to be sheared for health reasons generating about 150,000 tons of wool fiber per year, but in Europe about 75% of sheep wool fiber production is discharged because it is not suitable for the textile industry [25,26]. These fibers are quite resistant to degradation due to the particular structure of keratin and their management as special waste requires demanding treatments before disposal [25]. Sheep wool fibers can be used as reinforcement in cement mortars or for production of insulating panels with excellent hygrothermal and acoustic properties [25,27,28,29,30]. Sheep wool for this study was kindly provided by The Wool Company consortium. The material used for the test was stored in closed bags to avoid biological attack and was already washed with water and non-ionic detergent.

Lignin sulfonate is a lignin-based by-product of the paper and pulp production industry, from the separation of white cellulose and lignin. The worldwide annual production of lignin in pulp mills is around 50 million tons [31]. The final product is highly soluble in water (>10,000 mg/L) and has many applications: an additive for concrete, a binder for graphite and activated carbon, a fertilizer agent, a component for dispersants, bricks, and adhesives [32]. Lignin sulfonate was selected for its interesting properties as a soil stabilizer, and it is used to enhance principally durability and mechanical properties [33,34,35]. The additive was kindly provided by Burgo Group (Mosaico S.r.l., Vicenza, Italy) with the commercial name of Bretax CRO2, calcium lignin sulfonate, as brown powders with a typical coffee smell, with a commercial value of around 0.55 EUR/kg.

Tannins are extracted from different plants and are mainly composed of phenols. Tannins are principally commercialized as natural antioxidants to enhance the properties of wines and as natural stabilization aid for beer production, but is also used as antifungal, anticorrosion, and antibacterial agents, as well as a base agent for natural glue production [36,37,38]. The use of tannins for earthen construction and soil stabilization presents some interesting results. It is principally used as a dispersant, able to enhance the mechanical strength and modify the rheology of the system [38,39,40,41,42,43,44,45]. The powder of tannin used in the present research was kindly provided by Silvateam S.p.a. (San Michele Mondovì CN, Italy) company and extracted from the quebracho tree, a typical plant of Argentina. It appears as a brown powder and it is completely miscible in water. This additive has very dark color and can be used to change the mixture primary color and create a darker shade of brown. Among all the additives tested, this one has the highest commercial value, around 2–3 EUR/kg.

Grape seed flour is a by-product of wine industry production, composed mainly of seeds and minor parts by stems and peels. The annual worldwide production of grapes reaches almost 70 million tons and about 80% are used for wine production, generating 20–25% of waste on the total weight of material [46,47]. The main components of grape pomace are vitamins, antioxidants, unsaturated lipids, sterol, phenolic compounds, and soluble and insoluble fibers. The presence of fibers, combined with phenolic compounds, is quite useful to evaluate the possibility of its utilization with earth-based material [48]. The material supplied was kindly provided by Soliani—Italcol S.p.a. company. It was dried grape seed flour from oil extraction through the use of hexane and it is classified as bio-fuel with a low commercial value (0.065 EUR/kg). The material was sieved to remove all the particles exceeding 2 mm.

### 2.3. Mix Composition with Natural Additives

The five different mixture formulations were prepared with the additives to obtain stabilized specimens to be compared with control samples. Lignin sulfonate, citrus pomace and grape-seed flour were mixed with the base mix composed of a 1:3 soil-to-sand ratio (MIX) with a proportion of 1% dry weight. The wool fibers were mixed with a proportion of 0.25% dry weight, optimizing the percentage of addition based on previous research [27,28]. In the case of lignin sulfonate, tannins and citrus pomace, the choice of 1% of addition was based on previous literature research, criteria of biological risk of using biodegradable material, study of comparability and probability to detect the interactions between clay and biopolymers [23,33,34,38,40,41,49,50]. Since grape-seed flour has not been studied in literature and its weight/volume proportion is similar to citrus pomace, both the additives were considered using the same criteria. Moreover, the addition of 1 wt% was chosen for all the additives except wool, to limit the quantity of biodegradable material used while maintaining a reasonable probability of modifying the properties of the material and detecting its presence at a microscopic level.

The mixtures were prepared using a blender for 5 min to obtain a good and comparable level of mixing for all the mixtures but limiting the time to avoid excessive water evaporation. Distilled water was added up to optimal water content by pouring continuously during the mixing procedure. The Modified Proctor compaction test (Section 2.4) was used to determine the optimal water content [11]. The mixtures were left for 24 h in sealed containers to ensure uniform distribution of water before starting the sample preparation step. To allow easier discussion, the stabilized mixtures are called LIG (MIX + lignin sulfonate), TAN (MIX + tannins), WOOL (MIX + sheep wool fibers), GRA (MIX + grape-seed flour), CIT (MIX + citrus pomace).

### 2.4. Proctor Test and Samples Manufacturing Process

**Figure 2:**
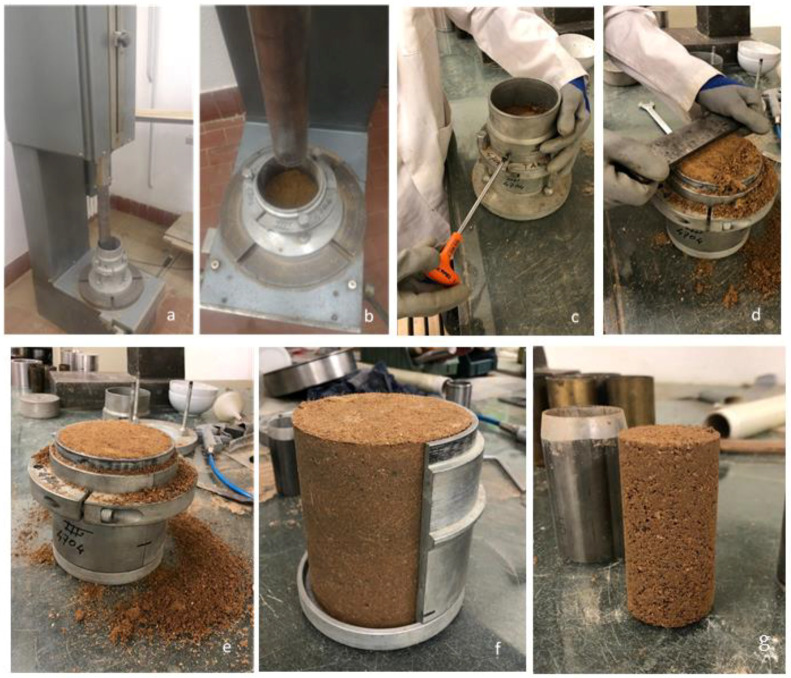
(**a**,**b**) The Proctor machine, the hammer applies blows to the mold; (**c**) the opening of the mold; (**d**) leveling of the top; (**d**,**e**) the proctor sample leveled; (**f**) opening the mold (**g**) sample cored after the extraction.

The Modified Proctor Test (MPT) was performed according to ASTM D1557 [51], to simulate a compaction effort similar to that given by pneumatic placement in RE construction [9,10]. Specimens were manufactured in five layers of equal mass and each layer was hit by the hammer 25 times with the compaction effort of 2703 kN∙m/m^3^ by using a proctor machine (Figure 2a,b). The constant compaction effort is given by the weight of the piston which falls from a defined level, as described by the standard ASTM D1557 [51]. The standardized mold dimensions were 10.24 cm in diameter and 12.50 cm in height (Figure 2c–f). By weighing the wet initial mass of the samples and the dry mass after oven drying at 105 °C for 24 h, it was possible to determine the actual amount of water in the soil and calculate the dry density and the water content of the sample. The drying procedure was applied following the guidelines for RE testing procedures on Proctor test protocol designed by Beckett at the University of Western Australia, compatible with the standard ISO/TS 17892 [52,53]. The maximum dry density value is obtained by repeating the test with different water contents. The optimum water content is assumed to be the one giving the maximum dry density. The compaction test was repeated so that the optimum water content could be determined for each type of mixture. The values obtained are reported in Section 3.1 and were used in all the following tests.

The samples for testing the mechanical properties were prepared by coring a smaller cylinder from a bigger proctor sample just after compaction, prepared at its optimum water content with the procedures illustrated in Section 2.4. This method was preferred to other techniques of preparation because it provides similar conditions to real RE walls, given by the use of dynamic compaction and superimposed layers of soil. In addition, the wet state of the samples facilitates the coring procedure.

A slenderness ratio of 2 (3.8 cm in diameter and 7.6 cm in height) was chosen to respect the guidelines for rammed earth and soil mechanics for UCS [19,54,55]. The size of the samples respects the criteria for the international ASTM standard of geotechnics D2166 and the State-of-the-Art Report of the RILEM TC 274-TCE (2021) on Testing and Characterization of Earth-based Building Materials and Elements [18,55].

From the Proctor sample of 10.24 cm in diameter it was possible to core up to two specimens of 3.8 cm in diameter, using a hand-driven load frame and a sharpened sampler (Figure 2g). The remaining soil of the Proctor sample was placed in the oven to dry for 24 h at 105 °C [52,53] to verify the final moisture content. The specimens for the UCS test were then conditioned at 20 °C and 59.14% RH, using a saturated solution of sodium bromide salt. Approximately 2 months were necessary to dry the samples and achieve the equilibrium of conditioning, estimated from the relative weight changes from 3 consecutive days smaller than 0.05%. A minimum of three samples for each different mixture was tested and the good reproducibility of the samples was ensured by standard deviation values lower than 5%, as suggested by the “*Guide de bonnes pratiques de la construction en terre crue*” [4].

### 2.5. Unconfined Compressive Strength Test

**Figure 3:**
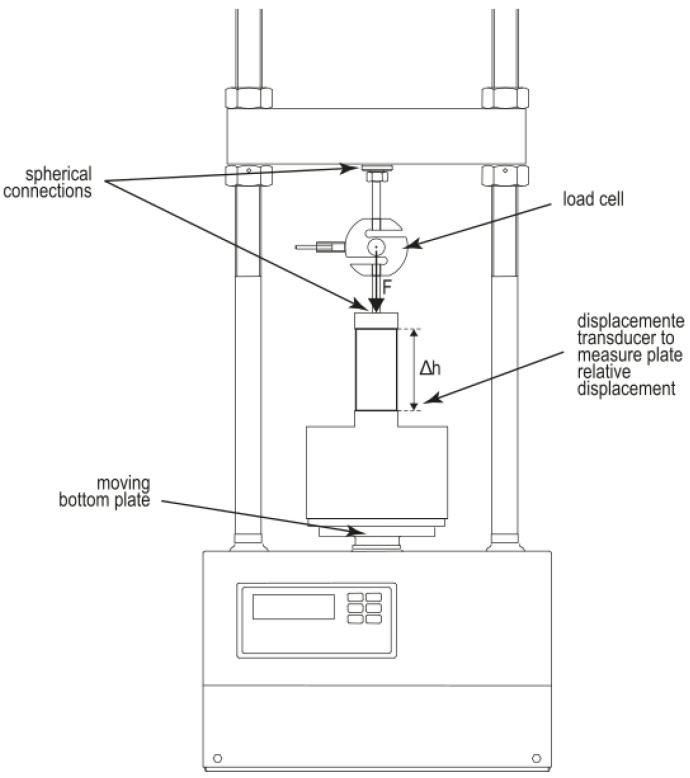
Schematic representation of the UCS test equipment, load frame and sensors.

Unconfined compressive strength was tested by applying a constant rate of vertical displacement and recording the corresponding load on the sample. Two steel plates on the top and the bottom of the specimens were connected with the load frame, while the lateral surfaces were left unconfined (Figure 3). The deformation was applied by the lower plate using a constant displacement rate, while a load cell was connected to the upper one to record the load until failure. A constant displacement rate of 1 mm/min was chosen to respect the ASTM D2166 criteria, which suggests applying the load to produce an axial strain at a rate of 0.5–2% [18]. This speed made it possible to perform the test in less than 5 min, until reaching the failure phase when the test was stopped and deformation was observed (Figure 3). The short duration of the test maintained a constant water content in the samples, as given by the initial conditioning. The load cell recorded the applied load, while a high precision displacement transducer was used to measure the vertical displacement of the sample to determine the axial deformation (Figure 3). All samples were measured by an electronic micrometer comparator at the beginning and end of the test, then dried in the oven for 24 h at 105 °C to verify the moisture content [52,53]. The test was executed the geotechnics laboratory LPM of Politecnico of Milan, Italy.

### 2.6. Microstructural Characterization

Several microscopic analyses tests were planned to investigate and explain the interactions between the earth and the additives that are responsible for the macroscopic behavior. In particular, the pore size distribution was investigated using mercury intrusion porosimetry (MIP) and the nitrogen adsorption isotherm. MIP made it possible to investigate the larger pore diameters, while the nitrogen isotherm completed the analysis on the smaller porosity and measured the specific surface area. The X-ray diffraction (XRD) test was used to verify the possible intercalation of the powder additives (lignin sulfonate and tannins) in the swelling clay lattice. Similarly, a scanning electron microscope (SEM) was used to characterize the surface of the additives and the samples. All the samples analyzed were prepared with the same methodology described in Section 2.4 and crushed to fine powders for the SEM, MIP and nitrogen sorption tests.

#### 2.6.1. Mercury Intrusion Porosimetry

Mercury intrusion porosimetry (MIP) is a method used to determine the dimensions of the pores and their distribution [56]. It takes advantage of a non-wetting liquid (mercury) that is forced into the pores by external pressure, which increases gradually until mercury reaches the smallest pores. Based on the knowledge of the volume intruded, the pore size distribution is determined at each pressure increment. This method is used to measure the range of pore entrance between 100 μm and 0.0025 μm [56]. For the analysis, an AutoPore IV 9500 porosimeter from Micromeritics Instrument Corporation, capable of applying pressures up to 207 MPa, was used. The contact angle between the solid and the mercury was assumed about 130° with a surface tension of 0.485 N/m. The quantity investigated for a typical experiment was around 0.5 g per sample.

#### 2.6.2. Nitrogen Adsorption–Desorption Isotherms

This method quantifies the pore volume and pore size distribution from the data of the nitrogen isotherms. The evaluation of the specific surface area from the nitrogen isotherm is based on the Brunauer, Emmett and Teller (BET) method [57]. This method is based on the assumption that the quantity of gas absorbed (through the monolayer, multilayer and capillary condensation) is related to the surface area and the porous structure of the material [58]. The test was performed using a TriStar 3000 analyzer from Micromeritics Instrument Corp. (Micromeritics Instrument Corporation, 2016, Norcross, GA, USA). The sorption/desorption isotherm was measured at 77.35 K on a typical sample of mass equal to 0.5 g. The data were studied with Hasley and Fass’s BJH model (0.00 face correction). The pore size distribution was determined using the Barrett–Joyner–Halenda (BJH) model and the evaluation of the specific surface area was based on the Brunauer, Emmett and Teller (BET) method [57].

#### 2.6.3. Scanning Electron Microscopy

The scanning electron microscope (SEM) allows the analysis of the surface of solid samples by using a focused beam of high-energy electrons. The signals generated by the interactions between the beam and the samples can be acquired to obtain tomographic (secondary electrons) or compositional (backscattered electrons) information with high spatial resolution [59]. A Cambridge Stereoscan 360 Electron Microscope was used for the observation of the samples, with an election high tension (ETH) of 20 kV and a working distance (WD) between 14 and 18 mm. A tungsten source was used to reveal a secondary electron signal. The samples were analyzed on the internal fracture surfaces, after being coated with a few nanometers of gold to prevent charging effects. In this procedure, it is difficult to obtain a flat surface for observation while preserving the original microstructure of the sample; consequently, it is difficult to maintain the focus, especially at high magnification [60].

#### 2.6.4. X-ray Powder Diffraction

X-ray powder diffraction (XRD) was used to characterize the crystalline structure and the mineral composition of the specimens before and after the addition of tannins and lignin sulfonate to detect the possible formation of new phases. The samples are powder samples prepared by crushing small fractions of specimens into an agate mortar. A diffractometer D8 Bruker Advance was used for the measurements, with the scansion speed at 1 s/step, with steps equal to 0.02° 2θ and a scansion range of 5–70° 2θ. 

## 3. Results

### 3.1. Proctor Test

**Figure 4:**
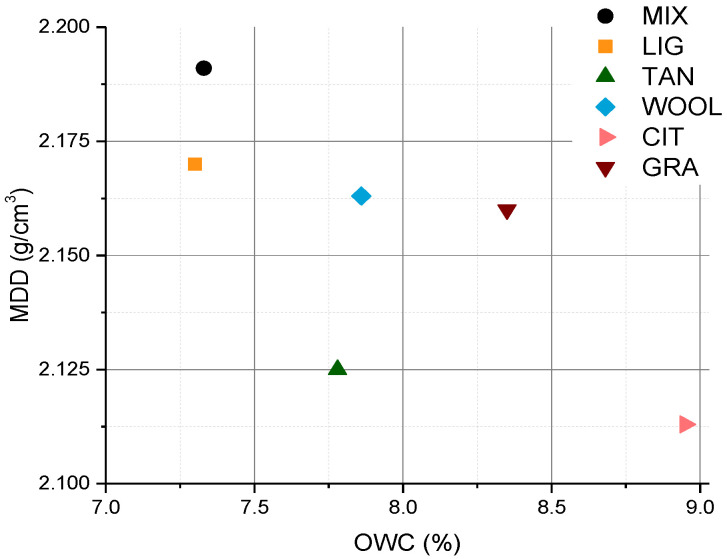
Optimum water content (OWC) versus the maximum dry density (MMD).

The maximum dry density values given by Proctor test results are between 2.19 and 2.11 g/cm^3^, within the highest range of RE density, as compared with the literature [10]. Figure 4 reports the data of the optimum water content (OWC, on the *x*-axis) and maximum dry density (MDD, on the *y*-axis) for stabilized and unstabilized samples, which are considered the control samples. The maximum dry density decreased for all the stabilized mixtures, while the optimum water content increased for all the stabilized mixtures compared to the control samples. This fact is probably due to the different modifications induced by the presence of additives. The presence of fibers with lower density than the soil grains reduced the dry density of the mixture, in accordance with the results reported by Galan-Marin et al. for sheep wool fibers [27]. Lignin sulfonate and tannins are soluble and did not modify the specific density of the material, but their presence prevented the reaching of the higher level of MIX compaction, indicating that the additives may increase the friction between the grains and favor their aggregation. Similar considerations are reported by Cai et al. and Arkema, respectively, for lignin sulfonate and tannin [38,49].

### 3.2. Mercury Intrusion Porosimetry

Different classifications of pore-size distribution exist in the literature. IUPAC proposes a largely used pore classification, but is not well adapted to soil media [61]. For this reason, a classification given by Romero [62] for soil geomechanical analysis was adopted to define five different ranges of porosity: macroporosity (pores size > 1 μm), mesoporosity (between 1 μm and 0.1 μm), microporosity (between 0.1 μm and 0.02 μm), and nanoporosity (>0.02 μm). The macropores were further divided into microcapillary pores (10–1 μm) and capillary pores (100–10 μm).

**Figure 5:**
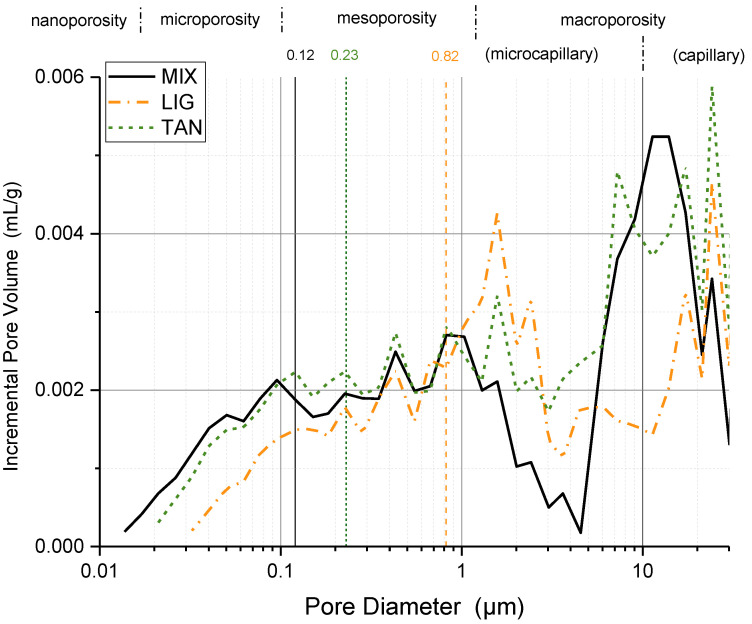
Incremental pore distribution from MIP analysis for the control samples and the LIG and TAN specimens.

**Figure 6:**
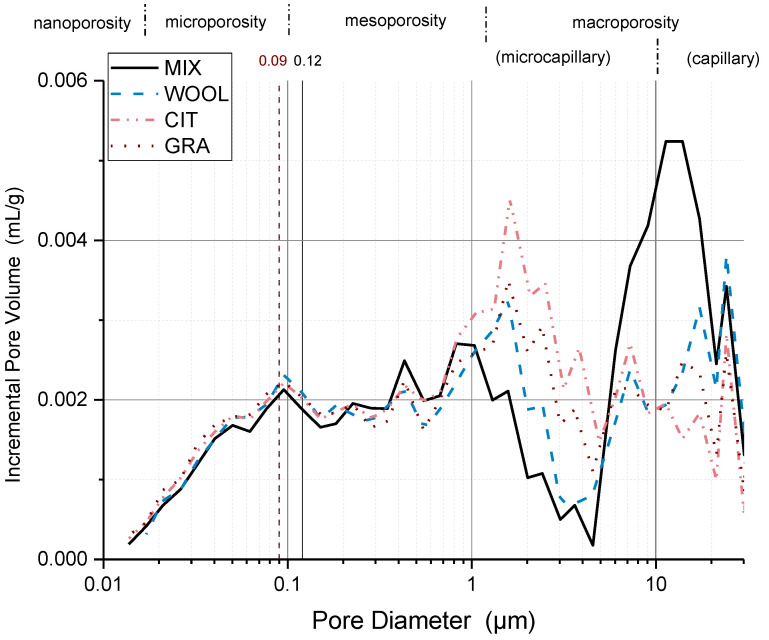
Incremental pore distribution from MIP analysis for the control samples (MIX) and the WOOL, CIT, and GRA specimens.

Figure 5 and Figure 6 provide a precise comparison of the samples stabilized with powder additives and fibers with the control samples (MIX).

The use of additives shows a major impact on the macroporosity range, probably due to the different effects of the additives during the compaction phase, which created a higher or lower friction state between grains. All the additives except tannins reduced the peak located around 10 μm within the capillary range. At the same time, the peak around 1.15 μm within the microcapillary range was intensified.

The compaction of clayish material generally presents a bimodal distribution, with two regions defined by the intra-porosity inside the aggregates and the inter-porosity, i.e., larger void structures between the aggregates. As can be observed in Figure 5 and Figure 6, the bimodal distribution cannot be clearly defined for specimens [63]. The high presence of sand in the mixture reduces the ability of clay to produce aggregates and the two typical peaks are distributed over a wider pore size range. Practically speaking, the material is more homogeneous and the pore distribution more uniform.

A first visual analysis of the results would place the division between the intra-aggregate and inter-aggregate regions, at around 5 μm for all the specimens. This division is not the same as would be determined following the protocol given by Romero et al. [64] and Bruno et al. [65], who analyzed the intrusion and extrusion cycles of MIP to identify the entrance pore diameter of the intra-aggregate region. 

Figure 5 and Figure 6 indicate the pore entrance diameter calculated with the latter technique for the different specimens compared with the control samples. The values were estimated at 0.12 μm for MIX and WOOL, while a value of 0.09 μm was estimated for GRA and CIT. As a first consideration, the use of fibers may not have influenced the entrance pore of the intra-aggregate region, while for LIG and TAN it was found to increase up to 0.82 and 0.23 μm, respectively.

Even if the incremental curve does not show a clear division of two peaks for MIP analysis, combining it with the nitrogen adsorption isotherm data (presented in Section 3.3) verified that there is a clear peak at around 0.1 μm that could explain the size of the entrance pore for intra-aggregate pores at around 0.12 μm. The value determined is consistent with the range of values identified by other studies on similar materials, between 0.01 and 3 μm [63,64].

Analyzing Figure 5, the pore size distribution of LIG compared to MIX seems to be reduced in the region of pores under 0.7 μm, in its intra-aggregate region (meso- and micropores) and increased in the inter-aggregate zone up to 5 μm (microcapillary zone), suggesting a total rearrangement of grains and pore filling using lignin sulfonate. The same behavior was observed by Zhang et al. [50]. Analyzing the incremental pore volume distribution of TAN, the peak around 10 μm in the macrocapillary zone is maintained, and the macro- and mesopore presence is increased, while only the micropore volume presents a small reduction compared to MIX. These data could suggest the formation of aggregates for TAN and in particular LIG [38,50]. As reported by Zhang [66], the use of lignin sulfonate tends to reduce the pore volume, due to a more stable and dense soil structure. Increasing the content of the additive (from 2 to 12%), the pores were filled and the bimodal distribution became a smooth unimodal distribution [66]. Due to the low content of lignin sulfonate in the present study, it is not possible to evidence a similar trend, but the reduction in the small porosity suggests the same behavior. Moreover, the peaks of the natural soil were shifted from around 5 μm towards a smaller pore diameter. It could be hypothesized that the peak of MIX located at 10 μm was also shifted to smaller pores: around 1 μm in the LIG specimens.

Considering Figure 6, the micro- and mesoporosity zone of the samples stabilized with the fibers, the porosity distribution peaks are unaltered for both intensity and position, suggesting that the presence of the fibers does not affect the smaller porosity of clay particles. The peak around 1.15 μm in the microcapillary zone is intensified, while the peak around 10 μm in the capillary zone is reduced. This behavior is more accentuated for CIT and GRA, which were added in higher quantity than WOOL. It should be noted that the porosity of the fibers is unknown; consequently, it is difficult to evaluate how their presence directly influences the specimens’ porosity value.

To conclude, considering the MIP results, the pore size distribution shows changes resulting from the addition of the additives. LIG pore distribution shows a reduction in the nano-, micro-, and mesopores in the intra-aggregate zone, while the inter-aggregate pore volume is increased. The peak around 10 μm in the macrocapillary pore zone is reduced for all the additives except TAN, while the peak around 1.15 μm in the microcapillary zone is intensified in the stabilized samples, in particular for LIG and CIT.

### 3.3. Nitrogen Adsorption–Desorption Isotherms

**Figure 7:**
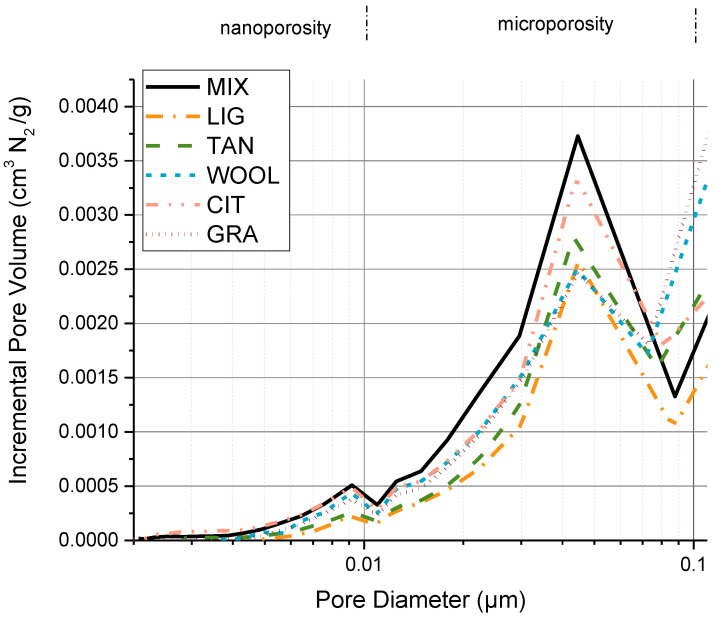
Incremental pore volume with respect to the pore diameter.

Figure 7 shows the incremental pore distribution obtained by the nitrogen sorption isotherm. All the specimens show a bimodal distribution with two peaks located at 0.009 μm and 0.045 μm. All the positions of the principal peak were maintained in the stabilized samples, with differences in the intensity of the peaks. The peak located at the nanoporosity scale is reduced in its intensity for the stabilized samples excluding WOOL, GRA, and CIT, suggesting that adding fibers does not modify the nanopore distribution. Similarly, the peak around 0.045 μm is reduced for all the additives except CIT.

**Figure 8:**
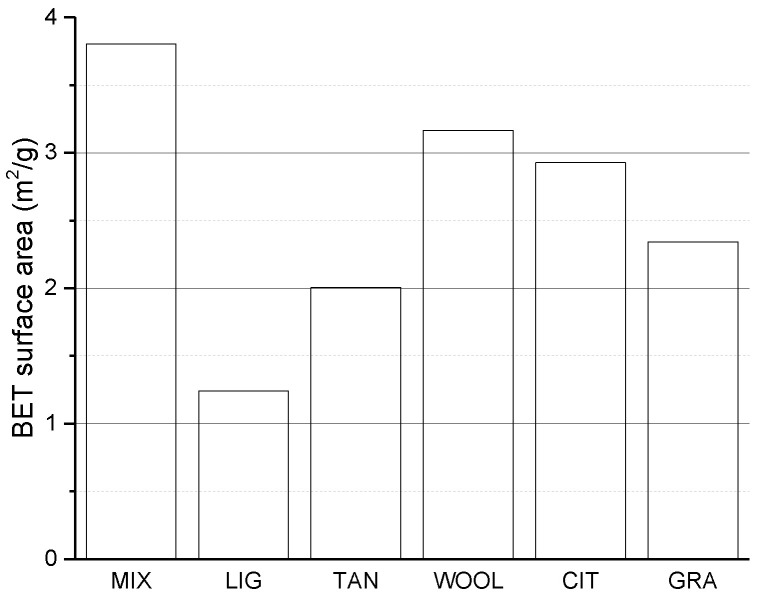
BET specific surface area for different samples.

The reduced porosity in the smaller pore diameter region could indicate that the formation of aggregates and pore-filling mechanism due to the additives reduces the microporosity for LIG and TAN. The values of specific surface areas confirm this hypothesis, in particular for LIG and TAN, which show a significant reduction for this value, as depicted in Figure 8. Moreover, the samples stabilized with the fibers show a decrease in their specific surface area, suggesting as a first hypothesis that the fibers can favor the aggregation of the material filling small pores, but with a lesser effect compared to lignin sulfonate and tannins. In addition, it should be noted that the specific surface area of the fibers alone was not estimated and compared with the soil’s surface area. Consequently, is more difficult to evaluate the reliability of these data for samples containing fibers.

To summarize the nitrogen sorption isotherm analysis, the pore volume in the nano- and micropore zone for LIG and TAN appears reduced, and the specific surface area shows a strong reduction, probably due to the additive’s ability to fill pores and aggregate soil particles. The use of fibers does not modify the nanopore distribution and only slightly reduces the specific surface area.

### 3.4. X-ray Diffraction

**Figure 9:**
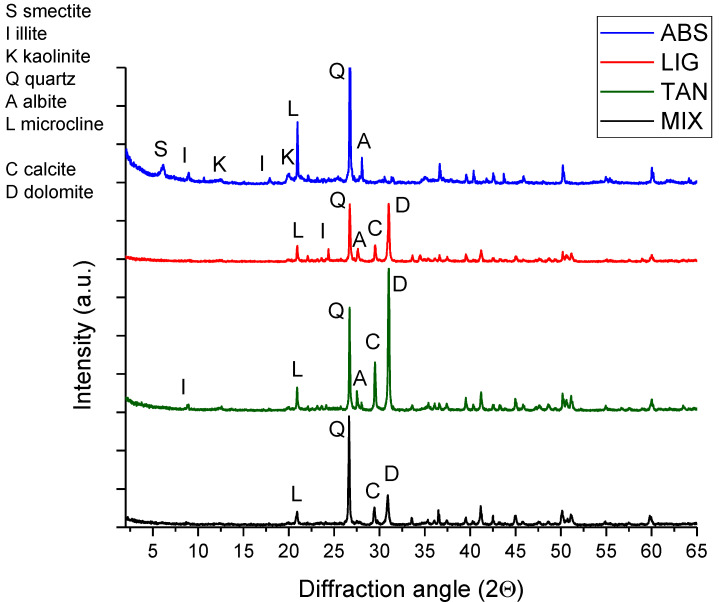
XRD diffraction pattern for ABS soil, MIX, and the stabilized mixtures: TAN and LIG.

Figure 9 reports the four diffraction patterns for the MIX, LIG, TAN and ABS powders. The analysis was not considered suitable for specimens with fibers (WOOL, CIT, and GRA) due to the larger dimensions of the fibers and their inability to interact with the mineral composition.

The peaks in the spectrum were identified using the Profex software databases (version 4.3.5) and the literature references in Poppe et al. [67]. The peaks in ABS diffraction patterns clearly showed the presence of smectite (6.01° 2θ), illite (9.0° 2θ, 17.88° 2θ, 24.44° 2θ), kaolin (12.4° 2θ, 19.8° 2θ), potassium feldspar (microcline 20.8° 2θ), albite (sodium feldspar 27.95° 2θ), and quartz (26.66° 2θ) [67].

In the diffraction patterns of MIX, LIG and TAN, calcite (29.1° 2θ) and dolomite (30.9° 2θ) are visible, due to the addition of calcareous sand to ABS [67]. Conversely, the clay peaks appear reduced or had disappeared as compared to ABS spectrum, because the high quantity of sand reduces the relative percentage of clay. The illite and albite peaks are still visible in the LIG and TAN patterns; conversely, they disappeared in MIX, probably due to the variability of the specimens and the low amount (a few milligrams) of soil used for the test.

In accordance with the results of the current study, Cai et al. [49] showed that the addition of 12 wt% lignin sulfonate to silty soil with non-swelling clays (10% of clay content) does not modify the presence and intensity of the peaks. Alazigha et al. [68] report the modification of the lignin sulfonate-treated soil (35% clay, with the presence of swelling clay), demonstrating the shifting of the peaks only for the swelling minerals, absent in Cai et al.’s study [49], which found that this phenomenon could suggest that lignin sulfonate connects the expansive clay minerals, but this was not verified in our study: due to the low clay content and the small amount of additive in the mixture, no significant difference in shifting was detected in the two spectra.

A small reduction in the peak intensity was observed in the LIG spectrum compared with MIX. The hypothesis that lignin sulfonate has a coating effect on soil particles could explain this phenomenon. As suggested by Al Alazigha et al. [68], the peaks of non-expansive clay minerals were reduced in their intensity in the spectrum of soil treated with lignin sulfonate, suggesting the external adsorption of the additive on these minerals, with the consequent reduction in the reflected incident ray and peak intensity. This possibility will be discussed in subsequent chapters, crossing different experimental results (Section 3.5).

Considering the spectrum of tannins, no shifting or reduction in peak intensity was observed. Conversely, Keita et al. [40] and Sorgho et al. [41] showed that the use of 1.44 wt% tannins reduces the peaks of illite, while the goethite and hematite peaks completely disappear, probably due to the formation of tannin macromolecular complexes with iron. No iron minerals were identified in the present study and the intensity of the illite peaks were not lower than MIX, while the position and presence of illite and albite were modified. As mentioned above, the small quantity of clay and the variability within the specimen may be responsible for the presence of albite and illite in the stabilized specimens and their absence in the control sample.

To conclude on the XRD results, the clay peaks are attenuated in the MIX spectrum compared with the ABS spectrum. The use of the additives does not significantly modify the spectra, and only the specimens stabilized with lignin sulfonate show a reduction in peak intensity, indicating that lignin sulfonate may have a coating effect on the soil particles, which reduces the intensity of reflection in the incident ray.

### 3.5. Scanning Electron Microscopy

#### 3.5.1. Additives and Stabilized Samples

**Figure 10:**
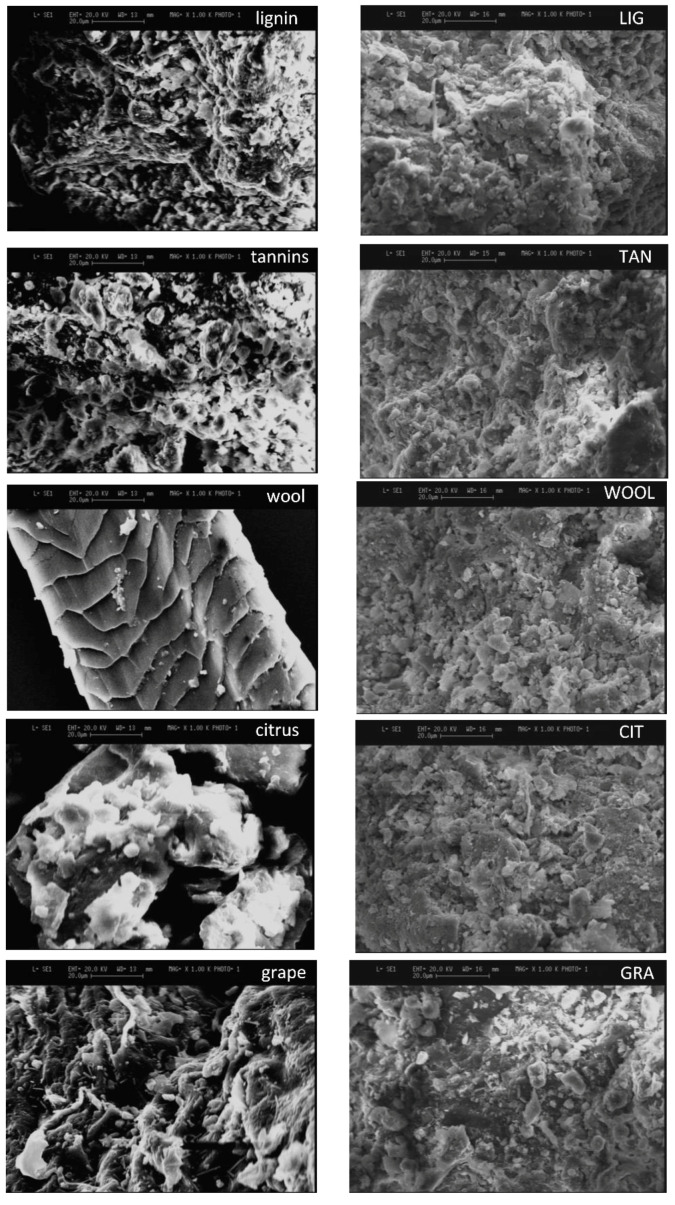
Comparison of the images of the pure additives and the specimens stabilized using the same additive, at the same magnification ×1000.

The images of the pure additives were investigated, so that their aspects can be easily identified. Figure 10 reports the images of the additives and the relative specimens stabilized with the same additive with magnification at ×1000. The appearance of lignin sulfonate and tannin powders is very similar, both composed of small particles and aggregates (visible particles from 20 to 1 μm). Wool fibers present a rough surface that could offer clay particles better adhesion. The diameter of the fiber can be estimated around 60 μm. Examples from the literature demonstrate the ability of wool particles to connect with other particles in a mixture with gypsum, making their surface completely covered by gypsum particles [25]. Hypothesizing a similar behavior with soil, the probability of identifying the fibers in stabilized samples is quite low. Citrus pomace and grape-seed flour present larger particle and pore dimensions than lignin sulfonate and tannins, which can reach up to 80 μm. As observed, due to the similarity between tannin, lignin sulfonate, citrus pomace, and grape-seed flour with the soil grains, it is very difficult to distinguish the presence of biopolymers in the stabilized specimens. In particular, lignin sulfonate and tannin should be able to dissolve in water, thus modifying the grain arrangement of the mixture. Moreover, due to the low quantity of additives in the mixtures, the probability of detecting their presence in the images is low. In addition, if the properties of the fibers allow them to connect with clay, the fibers are probably completely coated by the soil particles.

The samples here considered were dried in the oven at 105 °C due to the previous tests conducted on the specimens. The drying procedure could modify the porosity, due to pore shrinkage, making the structures between the different samples more uniform. However, the comparison between undisturbed specimens (presented in the next Section 3.5.2) and dried specimens showed no differences that suggest a reduction in larger pores; therefore, this hypothesis seems improbable. Moreover, this observation could depend on the portion of the sample investigated and not be representative of the whole sample.

#### 3.5.2. High Magnification of Samples Stabilized with Tannins and Lignin Sulfonate

**Figure 11:**
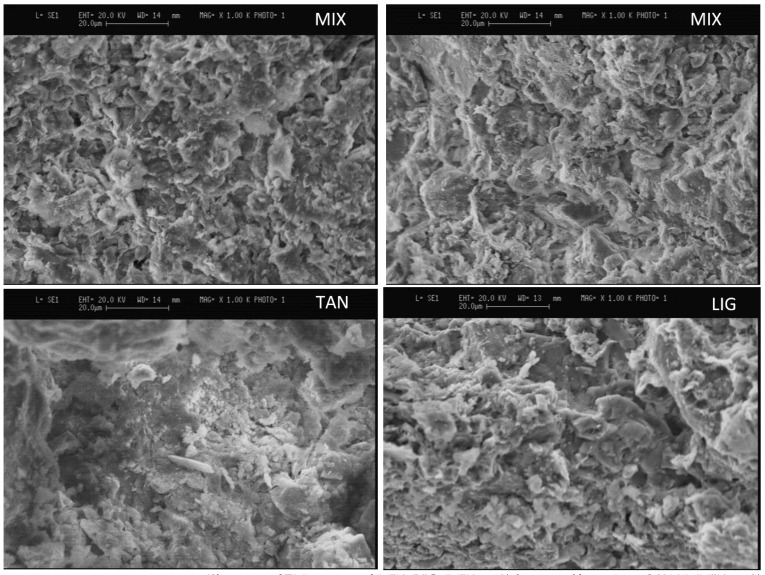
SEM images of MIX, LIG (MIX + 1% lignin sulfonate), and TAN (MIX + 1% tannins); magnification ×1000.

Specimens not subjected to the drying procedure were also analyzed with high magnification, to investigate the state of undisturbed samples with and without stabilization. High-magnification images of the undisturbed specimens stabilized with lignin sulfonate and tannins are compared with unstabilized specimens and examples of images from the literature. These two additives were selected for more detailed investigation because they are more easily distributed within the mixture given their powder nature, allowing for identify the possible interactions between particle surfaces and biopolymers. Figure 11 shows the comparison of the two different MIX specimens’ surface with TAN and LIG, magnified at ×1000. As a general observation, the MIX images present smaller particles and aggregates than TAN, while LIG shows an intermediate behavior. The smoother surface of TAN may suggest a cohesive mechanism and a filler of small porosity produced by the additive. Additional images magnified at ×2500 showed that the shape of clay particles is identified within the MIX specimens, with different orientations in space, while this is rarer for LIG and TAN images, suggesting that the additive can reorganize and bind clay and soil particles together. This result is in accordance with the data obtained on the specific surface area (Section 3.3). The reduction in the specific surface area for TAN and LIG specimens suggests a mechanism that reduces the available surfaces, for example filling pores, higher cohesion between the particles and formation of aggregates. This hypothesis for tannins is confirmed by literature results, demonstrating that they can bond clay and silt particles because of polymerization of the phenolic compounds in tannin when the mixture dries [38]. The SEM images reported by Arkema [38] show that the samples stabilized with tannin present a surface where the grains seem to be embedded in what appears like a paste, and the porosity is reduced compared to unstabilized specimens. In the study conducted by Arkema [38], TAN was added at a percentage of 2% (versus 1% tested in the present work), which probably helped to evidence the effects of the additive. Moreover, the higher presence of clay and silt particles gave a stronger coating effect as compared with MIX, which has larger soil grains as the main fraction. In conclusion, the smoother surface of TAN specimens and the reduction in the specific surface area are in accordance with literature results and suggest the cohesive effect of tannin, which reduces open porosity and rearranges the smaller particles with a binding mechanism.

The SEM images of specimens stabilized with lignin sulfonate presented a reduction in the visible clay particles and a possible reduction in small pores. The literature results showed a behavior for samples stabilized with lignin sulfonate that is similar to the tannin-stabilized samples. SEM images reported by Cai et al. [49] (silt treated with 12% lignin sulfonate) and Alazigha et al. (swelling clay soil with 2% lignin sulfonate) [68] show a reduction in small pores, increased interconnection between grains, reduction in visible clay particles, and a consequent coating effect on the grains. These studies suggest a possible interaction between soil and additive based on the additive adsorption onto soil particles and intercalation in the interlayer of expansive clay particles because of a hydrogen bonding and cation exchange mechanism. In the present study, the intercalation of lignin sulfonate in expansive clay particles was not shown by XRD, probably due to the lower content of additive and clays in MIX.

To conclude the observations on SEM analysis, the images of pure additives show smaller dimensions of lignin sulfonate and tannin particles and the aspect of grape-seed flour and citrus pomace, which is very similar to soil grains. The additives could not be precisely detected in the stabilized specimens, probably due to their similar aspects to soil and the possible good connection of the additives with clay particles that can completely coat the fibers and prevent their identification. Moreover, the low quantity of additives in the mixture reduces the probability of detecting the presence of the additives. For future analysis, the choice of soils with high clay content could facilitate the investigations of microscopic interactions between earth and biopolymers, although it is not recommended to be used for buildings materials due to the high risk of shrinkage.

The undisturbed specimens stabilized with lignin sulfonate and tannin show smoother surfaces with lower porosity for the TAN specimens, and the reduction in the visible clay particles with respect to the MIX images, suggesting a possible interaction and reorganization of the structure due to the presence of the additives. These data are in accordance with the reduction in the specific surface area for lignin sulfonate- and tannin-stabilized specimens. Considering the comparison with dried and undisturbed samples, there are no clear differences which suggest a reduction in larger pores.

### 3.6. Mechanical Characterization: Unconfined Compressive Strength Test

Table 2 reports the results of the UCS test, considering the maximum load the samples were able to undergo before failure. Experimental data reported are UCS values, relative improvement of UCS value compared to unstabilized control samples (MIX), the stiffness (E_c_), the density of the samples, the water content in the specimens (w) and the final saturation degree of the specimens at the end of the test (Sr__final_). The values are given as the average of a minimum of three specimens and the standard deviation is provided. The latter is around 0.5% for Sr__final_ and 0.80% for water content.

**Figure 12:**
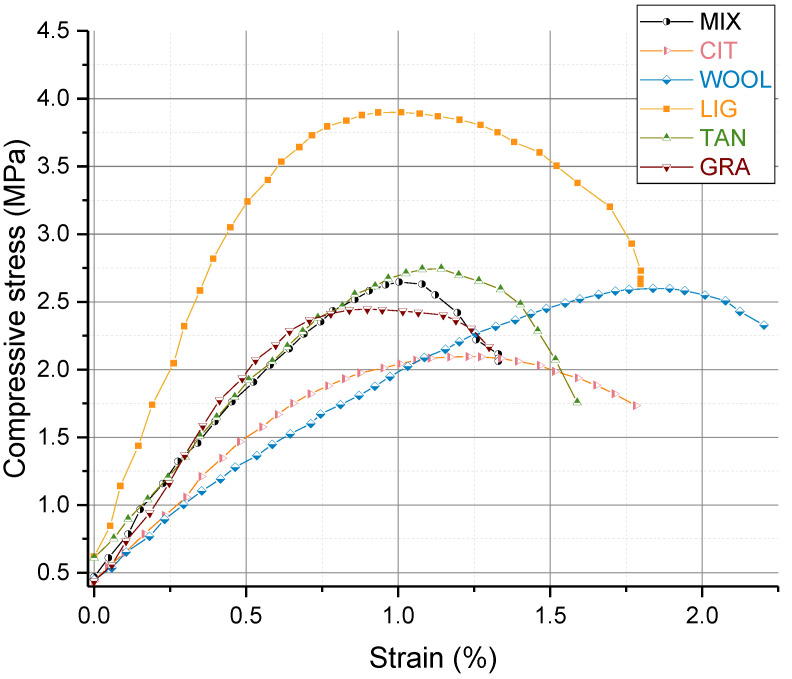
Stress–strain curves of different unstabilized and stabilized samples.

Experimental data are also reported in Figure 12 in terms of stress–strain curves, given by the following formulas:

(1) $$\varepsilon =\frac{\Delta h}{H_{0}}\;\cdot \;100,\;\sigma_{c}=\frac{F}{\;A}$$

where ε is the axial strain (%), Δh (mm) is the change in height of the specimens, H_0_ (mm) is the initial height of the samples, σ_c_ (MPa) is the compressive stress, F(kN) the applied load, and A (mm^2^) the corresponding average cross-sectional area.

Considering the stiffness, the slope of the linear part was calculated as the slope (E_c_) of the chord between 0.5 MPa and the 80% of the maximum load of the stress–strain curve, which identifies the linear behavior of the curve and represents a reasonable estimation of the stiffness of the material. The result given is the average of the E_c_ of the three samples. This method is an adaptation of the one proposed for concrete by Neville [69]. This technique is similar to the method suggested by Rodríguez-Mariscal et al. [70] for unfired earthen material, which considers the chord modulus as the slope of the secant line passing by one-third and two-thirds of the maximum load since the stress–strain curve shows a linear trend within this range [70]. The test was conducted on samples in equilibrium with the same temperature and humidity conditions (20 °C and 59.14%), in order to assure the comparability of the unsaturated conditions of each sample (same initial suction for all). The short duration (less than 5 min) makes reasonable to assume that all the water content changes are negligible during the test and therefore can be considered constant. Moreover, considering the Sr reported in Table 2, the relative variation of Sr during the UCS test is quite low, with values around 5%, suggesting that modification of the suction conditions is quite limited during the test. Under the hypothesis of the same initial conditions of suction and limited variations during the test, the differences in the unsaturated conditions of the specimens are expected to have a negligible influence on the UCS compared to the effect of the additives.

Figure 12 provides the UCS results in the stress–strain plane for all the stabilized specimens compared with the unstabilized condition (MIX). One representative curve has been selected for each type of sample. The use of the additives did not modify the typical behavior expected when working with soil aggregates. In the first part, the stress–strain curve rises up to about 80% of the maximum stress showing a linear relationship. The initial linear response seems to be prolonged at higher strain levels using wool fibers. In the following part, the stress–strain curve shows a decrease in the stiffness of the samples until the maximum stress is reached.

**Figure 13:**
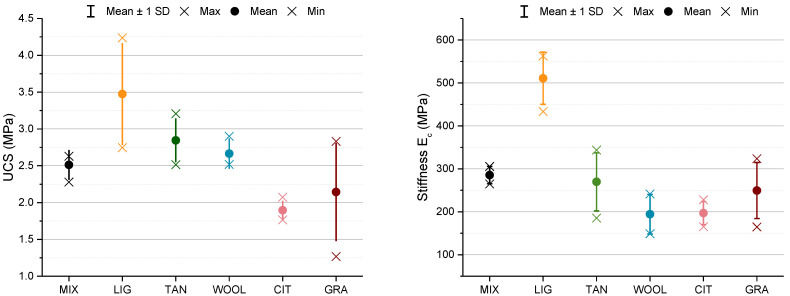
Unconfined compressive strength (UCS) and stiffness (chord modulus).

Figure 13 reports the results of UCS and E_c_ for the different specimens, comparing the average value with the minimum and maximum value recorded and the standard deviation. The unstabilized specimens of MIX showed UCS values around 2.5 MPa, while the UCS was reduced by about 25% when using citrus pomace and grape-seed flour. The use of wool, lignin sulfonate, and tannin increased the UCS by 6%, 38%, and 13%, respectively. Extensive discussion about UCS results is reported in the following sections, with additional considerations on the microstructural interaction and the mechanical behavior of the samples.

## 4. Discussion of UCS Based on Microscopic Characterization

As shown above, even a small amount of additives (1% at most) impacts material properties. Among the mixtures, LIG shows the maximum UCS and stiffness values. The 38% improvement of the UCS could be caused by additives due to the increased friction between the grains, which may be coated by the biopolymer, as evidenced by XRD and specific surface analysis.

The specific surface area showed the lowest value for the LIG and TAN samples, indicating the possible formation of aggregates that act by coating and bonding the smaller particles, which reduces their specific surface area. The LIG XRD analysis showed reduced intensity in the clay peaks, suggesting the possible interaction of the biopolymer with the surfaces of the particles, which reduces the intensity of the reflected X-ray. Moreover, the SEM images show a reduction in the visible clay particles for both LIG and TAN, confirming the previous hypothesis. These findings are in accordance with literature results [49,68], indicating that lignin sulfonate can connect clay particles, creating physical bonds between them and coating particle surfaces.

Considering TAN specimens, the increase in UCS is about 13%, while the stiffness of the material remained almost the same (Figure 13). The difference in the stiffness for LIG and TAN specimens could stem from the lower density of the TAN samples, which also maintain the macroporosity in the capillary region that is present in MIX, while LIG shows almost the same density as MIX, but the peak of the capillary pore region is reduced. The reduction in the macropores combined with the higher level of density could explain the increased stiffness and mechanical strength of the LIG samples. The same hypothesis of increased friction between the grains due to the coating and bonding effect of the additive can be considered. The information of the specific surface area and SEM images seems to confirm this hypothesis, in accordance with results presented by Arkema [38], who reports that tannin can connect grains and lock particles due to phenolic elements that can polymerize and adhere to soil particles.

Analyzing Figure 13, among the samples with fibers, there are differences in the peak stress: WOOL has a slight increase of 6.10%, while CIT and GRA have a similar decrease (24.5% and 24%, respectively). The stiffness was decreased by the use of fibers, especially for the CIT and WOOL samples. Literature results confirmed the behavior found for WOOL samples, showing an increase in UCS for a similar percentage of addition [27,29]. No literature references were found on citrus pomace and grape-seed flour fibers as reinforcement. Nevertheless, the literature available on the use of fibers shows that the differences in fiber characteristics can influence the results of the unconfined compressive strength. The lower UCS values recorded for GRA and CIT are probably due to the presence of the fibers that decrease the density of the samples, and consequently the stiffness. The increases in UCS values for the WOOL samples could be due to the longer fibers, which range from a few millimeters up to 10 cm, maintaining a small diameter. In this case, the long fibers seem to strengthen the structure, connecting the grains by locking them into the fiber network. Moreover, the wool fiber surface was shown to be a rough surface that could help the adhesion of clay on its surface (Section 3.5 on SEM images). In contrast, the reduced length of the fibers for GRA and CIT, under 2 mm, is probably not sufficient to create the same effect as wool fibers. Moreover, the higher quantity of addition (1% versus 0.25% wool addition) could enhance these phenomena, because the 1% addition is a compromise selected at the beginning of the study, which was not optimized to enhance the UCS. Optimizing the quantity of these fibers would have required extensive experimental work that did not come within the scope of the study.

The current study presents an investigation on a large number of biopolymers to identify the most promising additives. Before thinking to use this material on construction site, more solid and robust statistics to determine the mechanical properties should be presented. Moreover, the use of different types of soil and grain size distribution should be investigated further as far as the influence of these parameters on the mechanical behavior of the material is concerned. In addition, more investigations should be addressed to assess the effect of using different grain size distributions in combination with different samples’ dimensions.

Despite the reduced scale of RE samples, the values of UCS correspond to the range of acceptability for many international standards. The New Mexico code and Zimbabwe standard (SAZS 724:2001, 2001) are the most strict, up to 2 MPa, while New Zealand and Australia suggest minimum values around 0.5 MPa. The range suggested in the “*Guide des bonnes pratiques*” is between 0.9 and 1.7 MPa [4]. All the specimens tested in the present study reached a minimum UCS value equal to 1.5 MPa. Considering the most restrictive standards, citrus pomace and grape-seed flour should not be considered as suitable biopolymers for enhancing UCS, but their use for this aim is not completely excluded due to the possible investigation on the different quantities of materials that can be tested and the possibility of testing their effect on other soils.

## 5. Conclusions and Outlook

The present study showed that the use of lignin sulfonate, tannin and wool shows promising results to enhance the UCS of rammed earth. Summarizing the principal results of the UCS test, the use of lignin sulfonate and tannin increased the UCS value by 38% and 13%, respectively, compared to the control samples. Lignin sulfonate enhanced both the stiffness and UCS, probably due to the higher density in the specimens, the reduced macroporosity, and the possible coating effect of the activities on the particles, which seems to increase the effect of friction and particle locking. Moreover, the specific surface area is reduced, suggesting the formation of larger aggregates and a pore-filling mechanism.

The use of tannin increased the UCS values for stabilized specimens, probably due to a similar cohesive mechanism previously mentioned for lignin sulfonate. In this case, the stiffness shows values similar to MIX, probably due to the lower density of the specimens and the macropore maintained in the capillary region. Additionally, in this case the specific surface area is reduced, suggesting the formation of aggregates and a binding effect between the particles. In both cases, the formation of aggregates may favor particle locking and increase the UCS.

WOOL showed a UCS increase of about 6% and the stiffness of all the samples stabilized with fibers was reduced. This probably stems from the lower density of the samples provided by the fibers. Both citrus pomace and grape-seed flour decreased the UCS value in the stabilized samples by approximately 25%. The difference in the increased UCS of WOOL is probably due to the longer fibers with rough surface, which were able to better connect clay than citrus and grape-seed flour fibers.

Many aspects rise from the presented work that encourage further investigations. The mechanical characterization could be extended, in particular to evaluate the role of the fibers for enhancing the flexural and tensile strengths. The use of biopolymers, in particular powders, used in the soil stabilization field, is a promising direction for investigating the stabilization of RE materials. The powders showed easier mixing procedure, storage and processing than fibers, suggesting easier utilization on the construction site. At the same time, the use of fibers may have a lower environmental impact and be competitive when using a high quantity of material.

For future investigations, life cycle analysis could be considered to evaluate the sustainability of the use of the different additives compared with their advantages of processing and performances in unconfined compressive strength. As the last point, the evaluation of the impact of the use of the additives on the hygrothermal properties could be evaluated, both at the material and building scale.

## Figures and Tables

**Table 1:** Grain size distribution, specific gravity and Atterberg limits of soil (ABS), SAND, and MIX (1:3 soil-to-sand ratio). Liquid limit (LL), plastic limit (PL) and plasticity index (PI) are also reported [20]. Copyright 2021 Scientific.Net.

Earth	Clay (%)	Silt (%)	Sand (%)	Gs Specific Gravity	LL (%)	PL (%)	PI (%)
Diameter (mm)	d < 0.002	0.002 < d < 0.06	0.06 < d < 2				
ABS	36	44	20	2.65	42	24	18
SAND	-	4	96	2.78	-	-	-
MIX	9	12	79	2.77	24	18	6

**Table 2:** Results of the unconfined compression test, rate of UCS improvement, chord modulus E_c_, the water content in the specimens (w) and the final saturation degree in the samples. The data are reported as mean values ± their standard deviations.

Samples	Density (g/cm^3^)	UCS (MPa)	Improvement UCS (%)	Stiffness E_c_ (MPa)	w (%)	Sr__final_ (%)
MIX	2.084 ±0.032	2.51 ± 0.17	-	285 ± 17	0.73 ± 0.02	6.71 ± 0.69
LIG	2.086 ±0.027	3.47 ± 0.59	+ 38.3	511 ± 53	0.74 ± 0.01	6.64 ± 0.43
TAN	2.057 ±0.011	2.85 ±0.25	+13.3	270 ± 59	1.11 ± 0.02	9.36 ± 0.04
WOOL	2.069 ±0.014	2.67 ± 0.18	+ 6.1	194 ± 38	0.65 ± 0.03	5.89 ± 0.42
CIT	2.049 ±0.016	1.90 ± 0.11	−24.5	197 ± 23	0.76 ± 0.01	6.61 ± 0.34
GRA	2.054 ±0.044	2.14 ± 0.57	−14.7	250 ± 57	0.84 ± 0.04	7.85 ± 0.84

## Data Availability

The data reported in this article are available in the thesis defended the 13 December 2021 by Alessia Emanuela Losini, the title of the dissertation is *Rammed earth stabilization with waste or recycled materials and natural additives: characterization and simulation.*

## Abbreviations

RE	rammed earth
MIX	unstabilized RE specimens
LIG	MIX + lignin sulfonate
TAN	MIX + tannins
WOOL	MIX + sheep wool fibers
GRA	MIX + grape-seed flour
CIT	MIX + citrus pomace).
